# Differential proteomic analysis of lung tissues in rats with high-altitude pulmonary edema and screening of potential biomarkers

**DOI:** 10.3389/fphys.2026.1894550

**Published:** 2026-09-14

**Authors:** Dongmei Zhang, Tingting Liu, Hongfang Mu, Xiaolin Li, Chenyu Yang, Wenbin Li, Rong Wang

**Affiliations:** 1 Department of Pharmacy, The 940th Hospital of Joint Logistic Support Force, Lanzhou, China; 2 Department of Pulmonary and Critical Care Medicine, The Second Medical Center & National Clinical Research Center for Geriatric Diseases, Chinese PLA General Hospital, Beijing, China

**Keywords:** high-altitude pulmonary edema, hypobaric hypoxia, inflammatory response, lung proteomics, MMP9

## Abstract

**Introduction:**

High-altitude pulmonary edema (HAPE) is a severe complication of rapid exposure to high-altitude hypoxia, but its molecular mechanisms remain incompletely understood. This study aimed to characterize lung proteomic alterations in HAPE and identify candidate proteins associated with its pathophysiological processes.

**Methods:**

Male Wistar rats were randomly allocated to control and HAPE model groups (n = 20/group). The HAPE model was induced by exposure to simulated altitude (7,000 m) under hypobaric hypoxia combined with treadmill exercise for 48 h. Pulmonary edema was evaluated by lung wet-to-dry (W/D) weight ratio, bronchoalveolar lavage fluid (BALF) protein concentration, inflammatory cytokine measurement, and histopathological examination. Lung proteomic profiling was performed using liquid chromatography-tandem mass spectrometry (LC-MS/MS), followed by GO, KEGG, and PPI network analyses. Candidate proteins were further validated using Western blotting, immunohistochemistry, and RT-qPCR.

**Results:**

HAPE rats exhibited prominent pulmonary edema characterized by increased lung W/D ratios, elevated BALF protein leakage, enhanced production of inflammatory cytokines, and marked interstitial edema. Proteomic analysis identified 5,250 proteins, among which 288 DEPs were detected, including 160 upregulated and 128 downregulated proteins. Functional enrichment analysis revealed that these DEPs were primarily associated with immune and inflammatory responses, extracellular matrix organization, glycerophospholipid metabolism, phosphatidylinositol signaling, and cell adhesion pathways. Multi-level validation confirmed increased expression of MMP9, MMP8, Serpine1 (PAI-1), MPO, HK3, and MAP3K6 at both protein and transcriptional levels. PPI network analysis indicated that MMP9 represented a central hub protein with the highest interaction connectivity.

**Conclusion:**

This study provides a comprehensive proteomic profile of HAPE lung tissue and highlights the involvement of neutrophil-associated inflammation, extracellular matrix remodeling, fibrinolytic regulation, and metabolic adaptation in HAPE progression. The identified proteins, particularly MMP9, may serve as potential biomarkers and candidates for further mechanistic investigation.

## 1 Introduction

High-altitude pulmonary edema (HAPE) is a life-threatening form of altitude sickness caused by rapid ascent to altitudes above 2500 m, characterized by hypoxic pulmonary hypertension, increased vascular permeability, and intense inflammatory response (Eichstaedt et al., 2020; Hanaoka et al., 2024). Early clinical manifestations include exertional dyspnea, cough, fatigue, and headache, which may progress rapidly to resting dyspnea, cyanosis, hemoptysis, and in severe cases, respiratory failure and death (Hanaoka et al., 2024; Tetzlaff et al., 2022). With China’s vast highland territories and increasing numbers of individuals ascending to high altitudes for travel, work, or military operations, HAPE incidence continues to rise, posing a significant threat to public health and economic productivity (El Alam et al., 2022).

The pathogenesis of HAPE is multifactorial and incompletely understood. Current evidence supports contributions from hypoxic pulmonary vasoconstriction, energy metabolism imbalance, neuroendocrine alterations, fluid dysregulation, oxidative stress, and impaired alveolar fluid clearance (El Alam et al., 2022; Zubieta-Calleja and Zubieta-DeUrioste, 2021; Tenzing et al., 2024). Recent studies have further highlighted the critical roles of matrix metalloproteinases (MMPs), particularly MMP9, in degrading extracellular matrix (ECM) components and disrupting alveolar-capillary barrier integrity (Uysal and Uzun, 2019; Lo et al., 2018). Additionally, plasminogen activator inhibitor-1 (PAI-1/Serpine1) promotes pulmonary microthrombosis and fibrosis through TGF-β-mediated signaling (Shaikh et al., 2024), while myeloperoxidase (MPO)-driven oxidative stress and hexokinase-mediated glycolytic reprogramming further exacerbate hypoxic lung injury (Kargapolova et al., 2021; Lin et al., 2024). However, systematic investigations integrating these diverse molecular pathways at the proteome level remain limited.

Proteomics technology enables comprehensive, dynamic profiling of disease-related protein alterations at a global scale, offering a powerful approach to elucidate complex pathogenic mechanisms and discover novel therapeutic targets (Zhang et al., 2022). Unlike transcriptomics, proteomics directly reflects functional molecular changes in tissues and can reveal post-translational modifications, protein-protein interactions, and pathway activations that are critical to disease phenotypes. In the present study, we established a rat model of HAPE by combining simulated high-altitude hypobaric hypoxia with treadmill exercise, performed label-free quantitative proteomic analysis of lung tissues, and validated key candidate proteins by Western blotting, immunohistochemistry, and Reverse Transcription-Quantitative PCR (RT-qPCR). Our aim was to characterize the differential proteomic landscape of HAPE lung tissue and to screen potential biomarkers that may inform future mechanistic and translational research.

## 2 Materials and methods

### 2.1 Animals and HAPE model

Forty SPF-grade male Wistar rats (180–220 g) were purchased from Xi’an Nuoyou Biotechnology Co., Ltd. (License: SCXK (Xiang) 2019–0013). All animal procedures were approved by the Animal Ethics Committee of the 940th Hospital of Joint Logistic Support Force (approval No. 202211A027) and were conducted in accordance with institutional and national guidelines for the care and use of laboratory animals. Rats were housed at 23–25 °C and 40–70% humidity with free access to food and water. After 7 days of acclimatization, the rats were randomly divided into control and HAPE model groups (n = 20 per group).

Rats in the HAPE group were placed in a hypobaric hypoxic chamber for 48 h. The chamber was maintained at a barometric pressure of approximately 41 kPa (corresponding to a simulated altitude of 7,000 m) and was ventilated with ambient air (oxygen fraction ~20.9%; oxygen partial pressure ~8.6 kPa); thus, hypoxia resulted from the reduction in barometric pressure rather than in oxygen fraction. During the exposure, rats underwent treadmill exercise (12 m/min, 0.4 mA electrical stimulation, 20 min per session, twice daily). The general condition of the animals (activity, respiration, food and water intake, and body weight) was monitored daily. Humane endpoints were predefined: rats showing persistent lethargy, body weight loss exceeding 20%, or irreversible respiratory distress were euthanized immediately. Control rats were maintained at the local altitude (~1,500 m) without exercise or chamber exposure but received the same handling and husbandry procedures.

### 2.2 Sample collection and biochemical assays

After modeling, rats were anesthetized and sacrificed. The right lung was lavaged three times with 2 mL ice-cold 0.9% NaCl. The recovered bronchoalveolar lavage fluid (BALF) was centrifuged (1500 rpm, 15 min, 4 °C), and the supernatants were stored at −80 °C. The total protein concentration in BALF was determined using a bicinchoninic acid (BCA) protein assay kit. Lung tissue wet/dry weight ratio (W/D) was measured by drying the left upper lobe at 55 °C to constant weight. Inflammatory cytokines (IL-6, IL-1β, TNF-α) in BALF were detected by ELISA. Lung tissue sections were stained with hematoxylin-eosin (HE) for histopathological examination at 200× magnification.

### 2.3 Proteomic analysis

Lung tissue samples were lysed in buffer containing 8 M urea and 1% SDS. Protein samples (100 μg) were reduced with 10 mM TCEP (37 °C, 60 min), alkylated with 40 mM iodoacetamide (room temperature, dark, 40 min), and digested overnight with trypsin (1:50, w/w) at 37 °C. Peptides were desalted using HLB cartridges, dried by vacuum centrifugation, and reconstituted in 0.1% TFA.

LC-MS/MS analysis was performed on an EASY-nLC 1200 liquid chromatography system coupled to a timsTOF Pro2 mass spectrometer (Bruker, Germany). Peptides were separated on a C18 analytical column (75 μm × 25 cm; IonOpticks, USA). The mobile phase consisted of solvent A (2% acetonitrile and 0.1% formic acid in water) and solvent B (80% acetonitrile with 0.1% formic acid). A 60-min gradient was applied as follows: 0–45 min, 3%→28% B; 45–50 min, 28%→44% B; 50–55 min, 44%→90% B; 55–60 min, 90% B. The flow rate was 250 nL/min, and the electrospray voltage was set to 1.5 kV. Data were acquired in data-dependent acquisition (DDA) mode combined with parallel accumulation-serial fragmentation (PASEF; DDA-PASEF), with high-resolution time-of-flight (TOF) detection of peptide precursor ions and their fragment ions. The MS/MS scan range was 100–1,700 m/z.

### 2.4 Bioinformatics analysis

Raw LC–MS/MS data from (n = 3 rats per group) were processed and searched with Proteome Discoverer 2.2 against the Rattus norvegicus UniProt database. A false discovery rate (FDR) of <1% was applied at both the peptide and protein levels for identification. Data were normalized using median normalization. For missing-value handling, proteins with more than 50% missing values across samples were excluded, and the remaining missing values were imputed with half of the minimum detected value. All samples were analyzed in a single batch, and no significant batch effect was detected (as supported by the PCA of protein quantification reproducibility); therefore, no batch correction was required. Differential expression between the HAPE and control groups was assessed using Student’s t-test. Differentially expressed proteins (DEPs) were defined as follows: unadjusted P ≤ 0.05 with fold change (FC) ≥ 1.5 for upregulation, and unadjusted P ≤ 0.05 with FC ≤ 0.67 for downregulation. GO annotation was performed using InterProScan, KEGG pathway enrichment analysis was conducted for the DEPs, and PPI networks were constructed using the STRING database and visualized/analyzed for node degree to identify candidate hub proteins.

### 2.5 Western blot validation

Total protein was extracted from lung tissues, separated by SDS-PAGE, and transferred onto PVDF membranes. After blocking with 5% skim milk, the membranes were incubated with primary antibodies against MMP9, MMP8, Serpine1, MPO, HK3, and MAP3K6 at 4 °C overnight, followed by incubation with horseradish peroxidase (HRP)-conjugated secondary antibodies. Protein bands were visualized using enhanced chemiluminescence (ECL) reagent, and band intensities were quantified with ImageJ software. Relative protein expression levels were normalized to β-actin as the internal loading control.

### 2.6 Reverse transcription-quantitative PCR

Total RNA was extracted from 5–20 mg of lung tissue using RNA extraction reagent (Servicebio, China) with homogenization using zirconia beads on a three-dimensional cryogenic grinder (KZ-5F-3D). RNA was purified by chloroform substitute extraction, isopropanol precipitation, and 75% ethanol washing. RNA concentration and purity were assessed with a NanoDrop 2000 spectrophotometer (A260/A280 ratio between 1.8 and 2.0), and RNA was diluted to 200 ng/μL. Reverse transcription was performed using SweScript All-in-One RT SuperMix for qPCR (with gDNA Remover; Servicebio, cat. no. G3337) in a 20-μL reaction containing 4 μL SuperMix, 1 μL gDNA Remover, 10 μL total RNA, and nuclease-free water, with the program: 25 °C for 5 min, 42 °C for 30 min, and 85 °C for 5 s. qPCR was performed using 2× Universal Blue SYBR Green qPCR Master Mix (Servicebio, cat. no. G3326) on a Bio-Rad CFX Connect real-time PCR system in a 15-μL reaction containing 7.5 μL Master Mix, 1.5 μL primer mix (2.5 μM), 2.0 μL cDNA, and 4.0 μL nuclease-free water, with three technical replicates per sample. The cycling program was: 95 °C for 30 s; 40 cycles of 95 °C for 15 s and 60 °C for 30 s; followed by melting curve analysis from 65 °C to 95 °C with 0.5 °C increments. β-actin served as the internal reference gene, and relative mRNA expression was calculated using the 2^−ΔΔCt^ method. Primer sequences are listed in **Table 1**. Amplification and melting curves are provided as **Supplementary Figures S1**, **S2**.

**Table 1:** Primer sequences used for RT-qPCR (5′→3′).

Gene	Forward primer (5′→3′)	Reverse primer (5′→3′)
β-actin	TGCTATGTTGCCCTAGACTTCG	GTTGGCATAGAGGTCTTTACGG
MMP8	CCTTGCCCATGCCTTTCAAC	AGCCACGAGAAACAGGTTGT
MMP9	TCCAGCATCTGTATGGTCGTG	GCAGTGGGACACATAGTGGG
Serpine1	TCTTCCTCCACAGCCATTCTAGT	GGATTGTCTCTGTTGGATTGTGC
MPO	CTCAAGATCCCACCCAATGAC	TGCGAATGGTAATGTTGCTCC
HK3	CCATGTGAAGTCGGGCTTATTG	GCATCGTCGAAGGTGGTCAGTA
MAP3K6	GGCACAAATCCATACTCCTAACC	CAGCGAGTCCAACACGAACAT

### 2.7 Immunohistochemistry

Lung tissues were fixed in 4% paraformaldehyde, embedded in paraffin, and cut into 4-μm serial sections. After deparaffinization in xylene and rehydration through graded ethanol, antigen retrieval was performed in citrate buffer. Endogenous peroxidase activity was quenched with 3% H_2_O_2_, and non-specific binding was blocked with 5% bovine serum albumin (BSA) or normal goat serum for 30 min. Sections were then incubated overnight at 4 °C with the following primary antibodies: rabbit anti-HK3 (1:100, ProMab Biotechnologies, Cat# P07985), rabbit anti-MAP3K6 (1:200, Servicebio, Cat# GB13294), rabbit anti-MMP8 (1:150, ProMab Biotechnologies, Cat# P17266), rabbit anti-MMP9 (1:800, Servicebio, Cat# GB11132), mouse anti-MPO (1:2000, Servicebio, Cat# GB150177), and mouse anti-Serpine1 (1:500, ProMab Biotechnologies, Cat# 30306). After rewarming, sections were incubated with the corresponding S-vision polymeric secondary antibodies (goat anti-rabbit for HK3, MAP3K6, MMP8, and MMP9; goat anti-mouse for MPO and Serpine1) at room temperature for 30 min. Signals were developed with 3,3′-diaminobenzidine (DAB), counterstained with hematoxylin, differentiated with hydrochloric acid–alcohol, blued with ammonia water, dehydrated through graded ethanol, cleared in xylene, and mounted with neutral balsam. Phosphate-buffered saline (PBS) was used in place of the primary antibody as a negative control. Sections were observed and photographed under a light microscope (scale bar: 20 μm). Semi-quantitative analysis was performed using the histochemistry score (H-score), calculated as the product of staining intensity and the percentage of positive cells, with n = 3 per group. Statistical analysis was performed using GraphPad Prism 9.0.

## 3 Results

### 3.1 Establishment and evaluation of the HAPE rat model

Compared with the control group, the HAPE model group showed a significantly increased lung W/D ratio and BALF total protein concentration (P < 0.05; **Figures 1A, B**), indicating the development of pulmonary edema and increased alveolar-capillary barrier permeability. Histopathological examination revealed intact alveolar architecture in the control group, whereas the HAPE group exhibited interstitial pulmonary edema, swelling of alveolar epithelial cells, and pink proteinaceous exudate within the alveolar spaces (**Figures 1C, D**). In addition, the levels of the inflammatory cytokines IL-6, IL-1β, and TNF-α were significantly elevated in the HAPE group compared with the control group (P < 0.05; **Figure 1E**). These results confirmed the successful establishment of the HAPE rat model.

**Figure 1:**
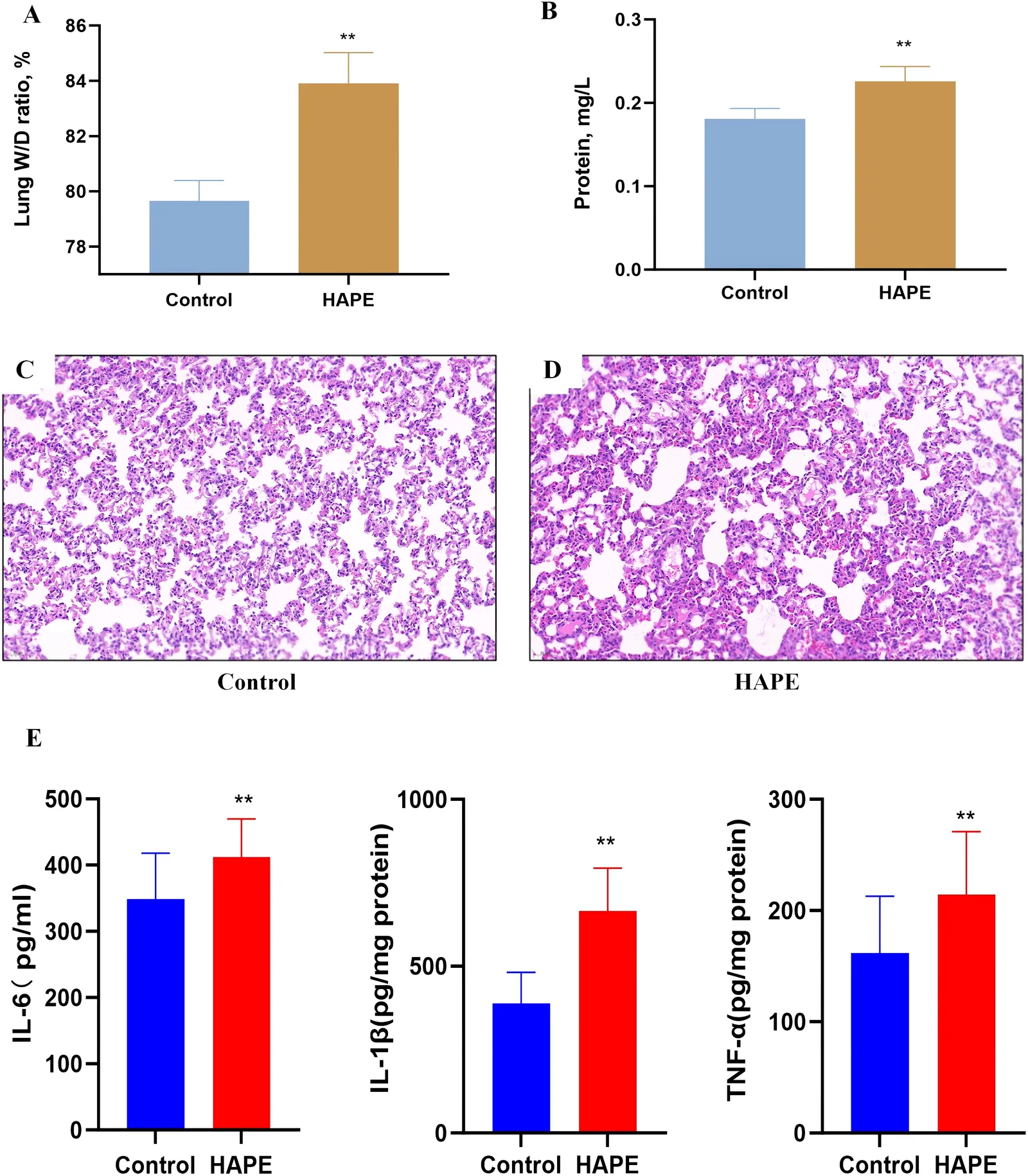
Establishment and evaluation of the HAPE rat model. **(A)** Lung wet-to-dry weight (W/D) ratio. **(B)** Total protein concentration in bronchoalveolar lavage fluid (BALF). **(C, D)** Representative hematoxylin and eosin (H&E)-stained lung sections from the control and HAPE groups at 200× magnification (n = 3 rats per group). **(E)** Levels of IL-6, IL-1β, and TNF-α in BALF measured by ELISA. Data in panels **(A, B, E)** are presented as mean ± SD (n = 20 rats per group). Comparisons between the two groups were performed using an unpaired two-tailed Student’s t-test. **P < 0.01 versus the control group.

### 3.2 Proteomic profiling and differential protein analysis

A total of 336,802 peptide-spectrum matches, 51,085 peptides, and 5,250 proteins were identified from the lung tissue samples. Three-dimensional principal component analysis (PCA) demonstrated clear separation between the control and HAPE groups (**Figure 2A**), and hierarchical clustering of DEPs revealed distinct expression patterns between the two groups (**Figure 2B**). Based on the screening criteria, a total of 288 DEPs were identified, of which 160 were upregulated and 128 were downregulated in the HAPE group. The volcano plot (**Figure 3**) illustrates the distribution of upregulated (160, red), downregulated (128, blue), and non-significantly changed proteins (4,962, gray). Notably, the key candidate proteins subsequently validated in this study—MMP9, MMP8, MPO, Serpine1 (PAI-1), HK3, and MAP3K6—were all significantly upregulated in the HAPE group. Representative DEPs are listed in **Supplementary Table 1**.

**Figure 2:**
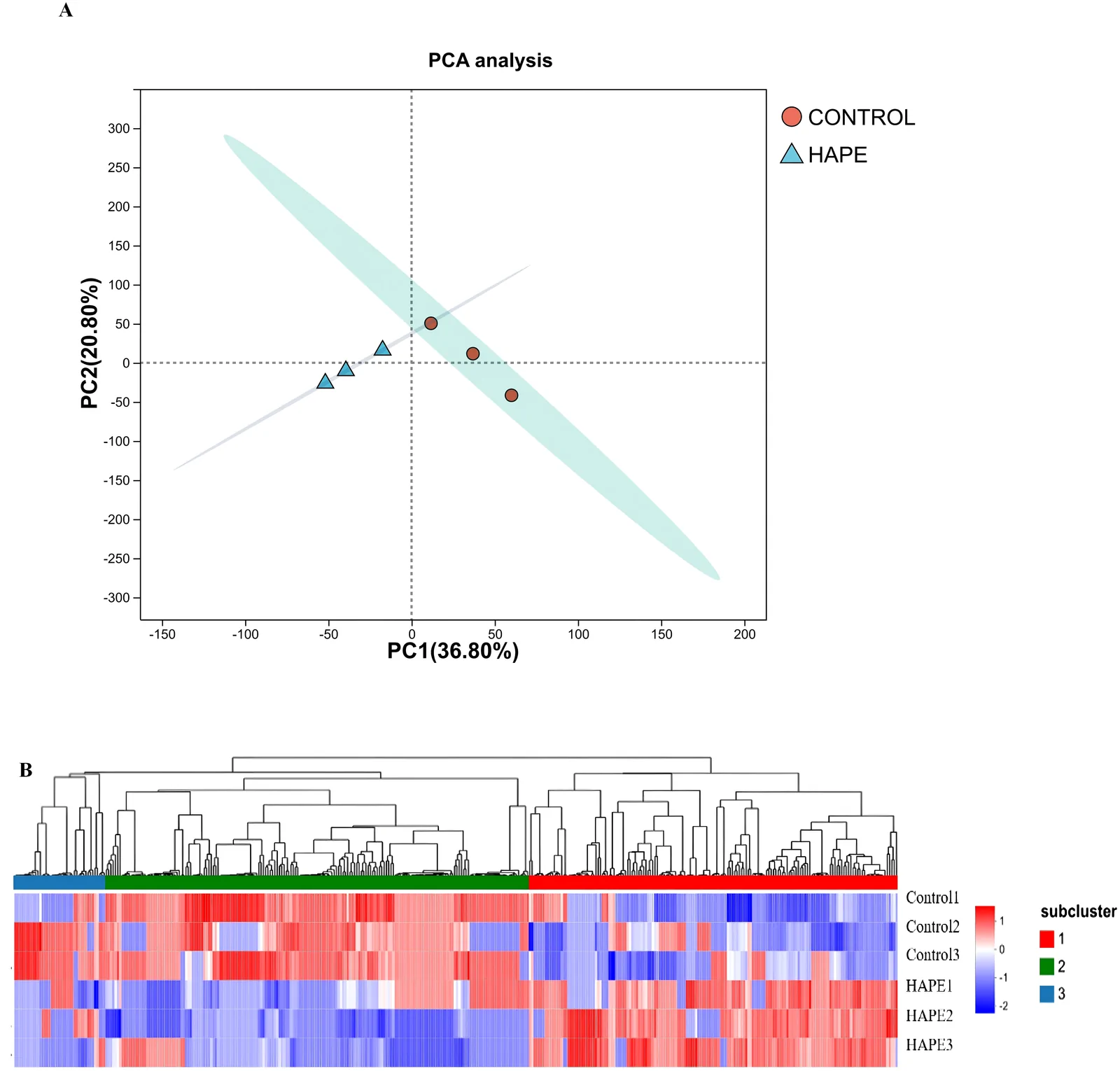
Overview of proteomic data. **(A)** three-dimensional principal component analysis (PCA) of the proteomic data; **(B)** hierarchical clustering heatmap of the differentially expressed proteins (DEPs). (n = 3 per group).

**Figure 3:**
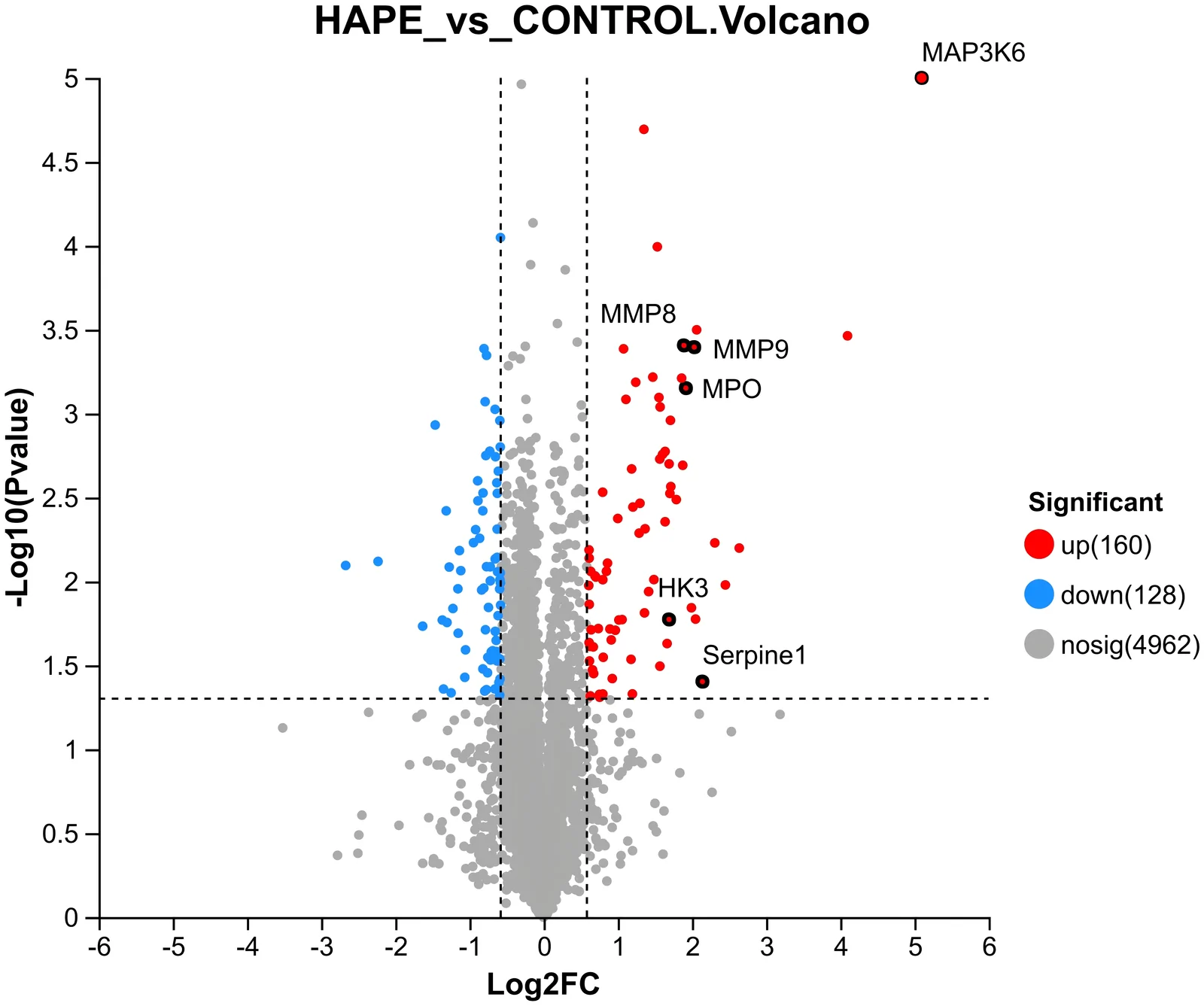
Volcano plot of differentially expressed proteins between the HAPE and control groups. The x-axis represents Log2 fold change (HAPE vs. control) and the y-axis represents −Log10 (P value). Upregulated proteins (n = 160) are shown in red, downregulated proteins (n = 128) in blue, and non-significantly changed proteins (n = 4,962) in gray. Key candidate proteins validated in this study (MMP9, MMP8, MPO, Serpine1, HK3, and MAP3K6) are annotated; all were significantly upregulated in the HAPE group. Screening criteria: P ≤ 0.05 with FC ≥ 1.5 (upregulated) or FC ≤ 0.67 (downregulated); unadjusted P values, unpaired two-tailed Student’s t-test (n = 3 per group).

### 3.3 GO enrichment and subcellular localization analyses

GO enrichment analysis of the DEPs was performed, and the top 20 enriched terms are shown in **Figure 4A**. These included 13 biological process (BP) terms, mainly related to immune and inflammatory responses—such as humoral immune response, immune response, defense response, regulation of immune system process, positive regulation of inflammatory response, positive regulation of cell activation, epithelial cell proliferation, acute inflammatory response, antibacterial humoral response, positive regulation of leukocyte activation, regulation of inflammatory response, and antimicrobial humoral response; 4 cellular component (CC) terms, including extracellular region, extracellular space, MHC class II protein complex, and MHC protein complex; and 3 molecular function (MF) terms, including DNA-binding transcription activator activity, heparin binding, and serine-type endopeptidase activity. Subcellular localization analysis (**Figure 4B**) indicated that the DEPs were predominantly distributed in lysosomes, the Golgi apparatus, the nucleus, peroxisomes, the cytoplasm, mitochondria, the extracellular matrix, the plasma membrane, and the endoplasmic reticulum.

**Figure 4:**
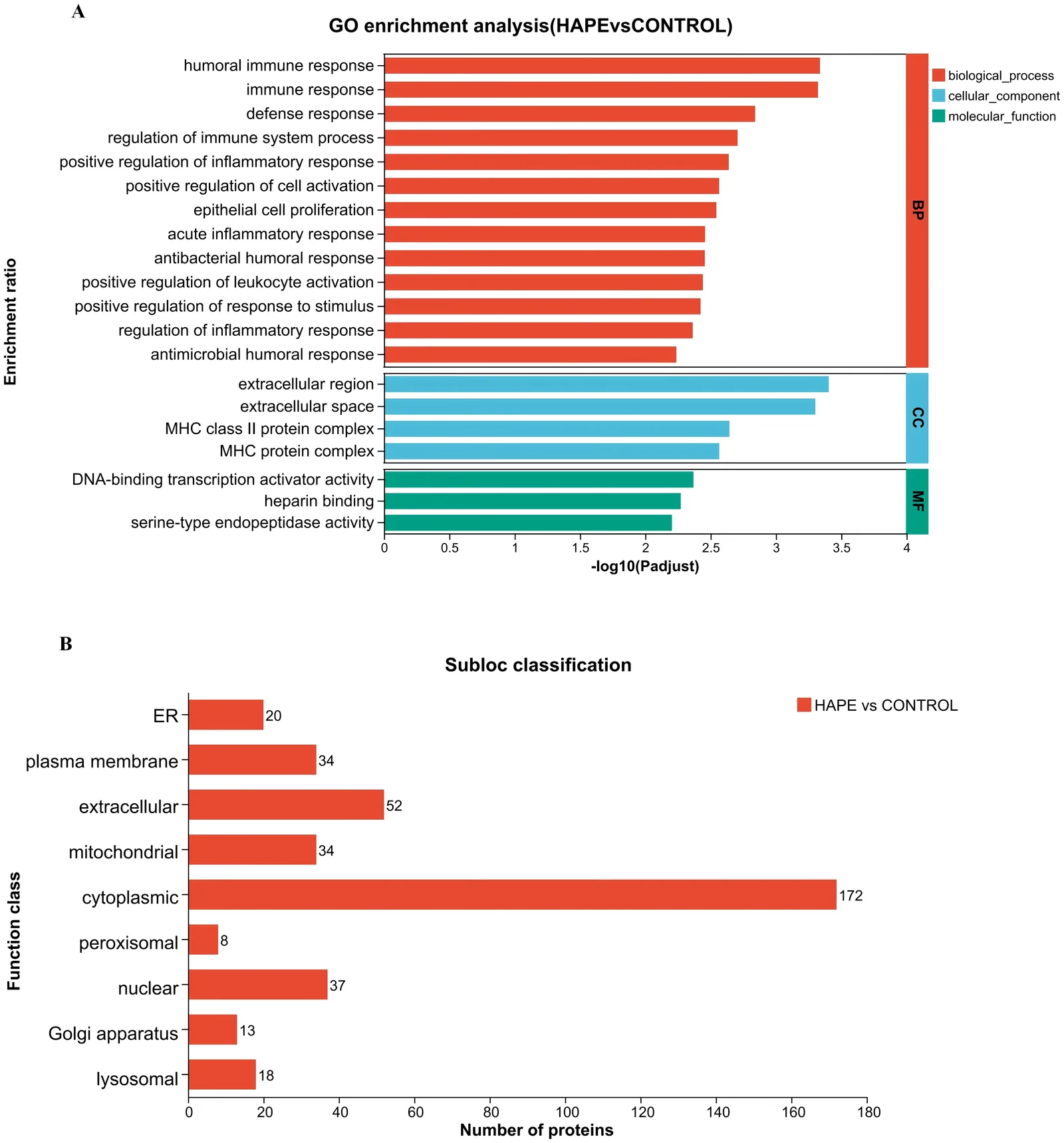
GO enrichment and subcellular localization analyses of the DEPs. **(A)** Top 20 enriched Gene Ontology (GO) terms, including 13 biological process (BP), 4 cellular component (CC), and 3 molecular function (MF) terms; **(B)** subcellular localization distribution of the DEPs. Enrichment analysis was performed using InterProScan-based annotation (n = 3 per group).

### 3.4 KEGG pathway enrichment analysis

KEGG pathway enrichment analysis identified 20 significantly enriched pathways (**Figure 5A**), including glycerophospholipid metabolism, the phosphatidylinositol signaling system, cell adhesion molecules, rheumatoid arthritis, antigen processing and presentation, systemic lupus erythematosus, and asthma. A chord diagram linking the enriched pathways with their associated DEPs is shown in **Figure 5B**. Eleven key proteins were identified within these pathways: MAP3K6, HSPA13, RGS3, MHC class II molecules, HK3, MPO, MMP9, CTSG, ITGAM, SLC2A3, and Serpine1.

**Figure 5:**
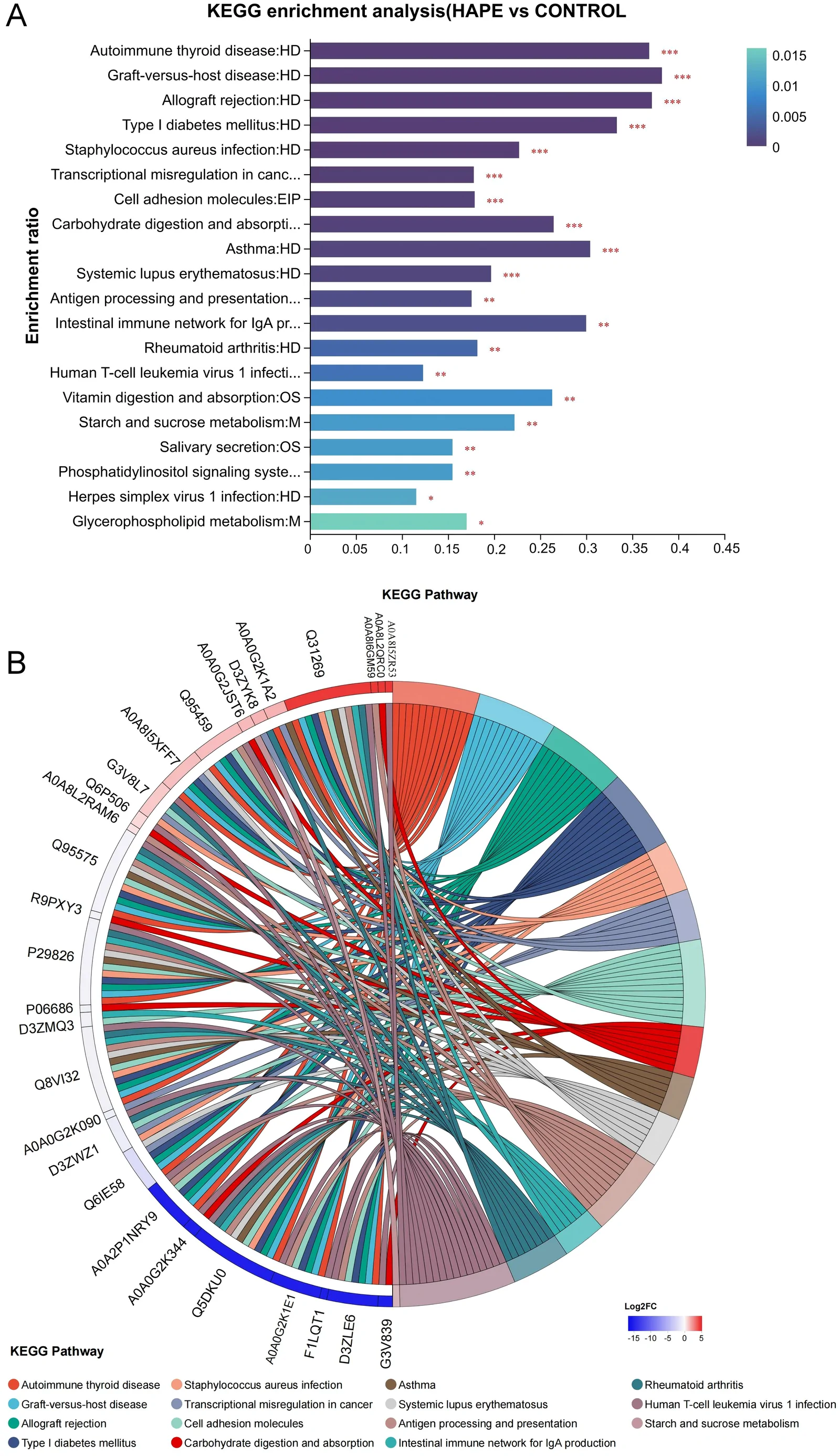
KEGG pathway enrichment analysis of the DEPs. **(A)** Bubble plot of the 20 significantly enriched KEGG pathways; **(B)** chord diagram showing the relationships between enriched pathways and their associated key DEPs (MAP3K6, HSPA13, RGS3, MHC class II, HK3, MPO, MMP9, CTSG, ITGAM, SLC2A3, and Serpine1) (n = 3 per group).

### 3.5 PPI network analysis

The PPI network of the DEPs comprised 81 nodes (proteins) and 384 interaction edges, with an average node degree of 9.48 as computed by the STRING database output (**Figure 6**). MMP9 exhibited the highest degree value and was thus identified as the central hub protein. MPO, Serpine1, and MMP8 also occupied hub positions within the network, whereas MAP3K6 and HK3 showed no direct interactions with the other hub proteins.

**Figure 6:**
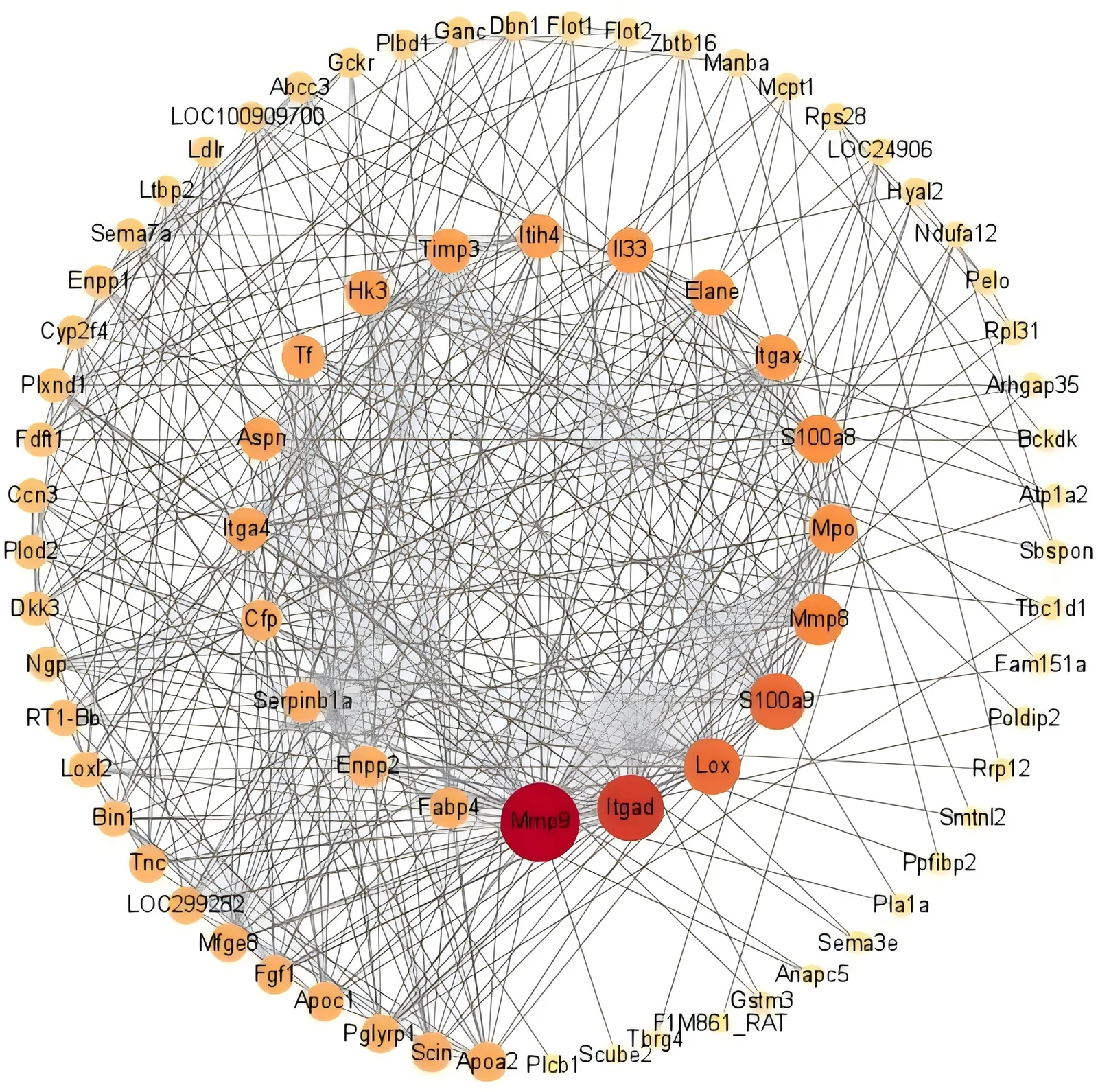
PPI network analysis. Protein-protein interaction (PPI) network of the DEPs (81 nodes, 384 interaction edges, average node degree 9.48) constructed using the STRING database; MMP9 exhibited the highest degree as the central hub, with MPO, Serpine1, and MMP8 also at hub positions.

### 3.6 Western blot validation

Six candidate proteins, namely MMP9, MMP8, Serpine1, MPO, HK3, and MAP3K6, were selected for validation by Western blotting. Consistent with the proteomic results, all six proteins were significantly upregulated in the lung tissues of the HAPE group compared with the control group (P < 0.05; **Figures 7A, B**). It should be noted that the key DEPs selected for validation were prioritized based on their central positions in the KEGG enrichment and PPI network analyses, and all of them were upregulated; validation of downregulated DEPs was therefore not included in this study and is acknowledged as a limitation (see Limitations and Future Directions).

**Figure 7:**
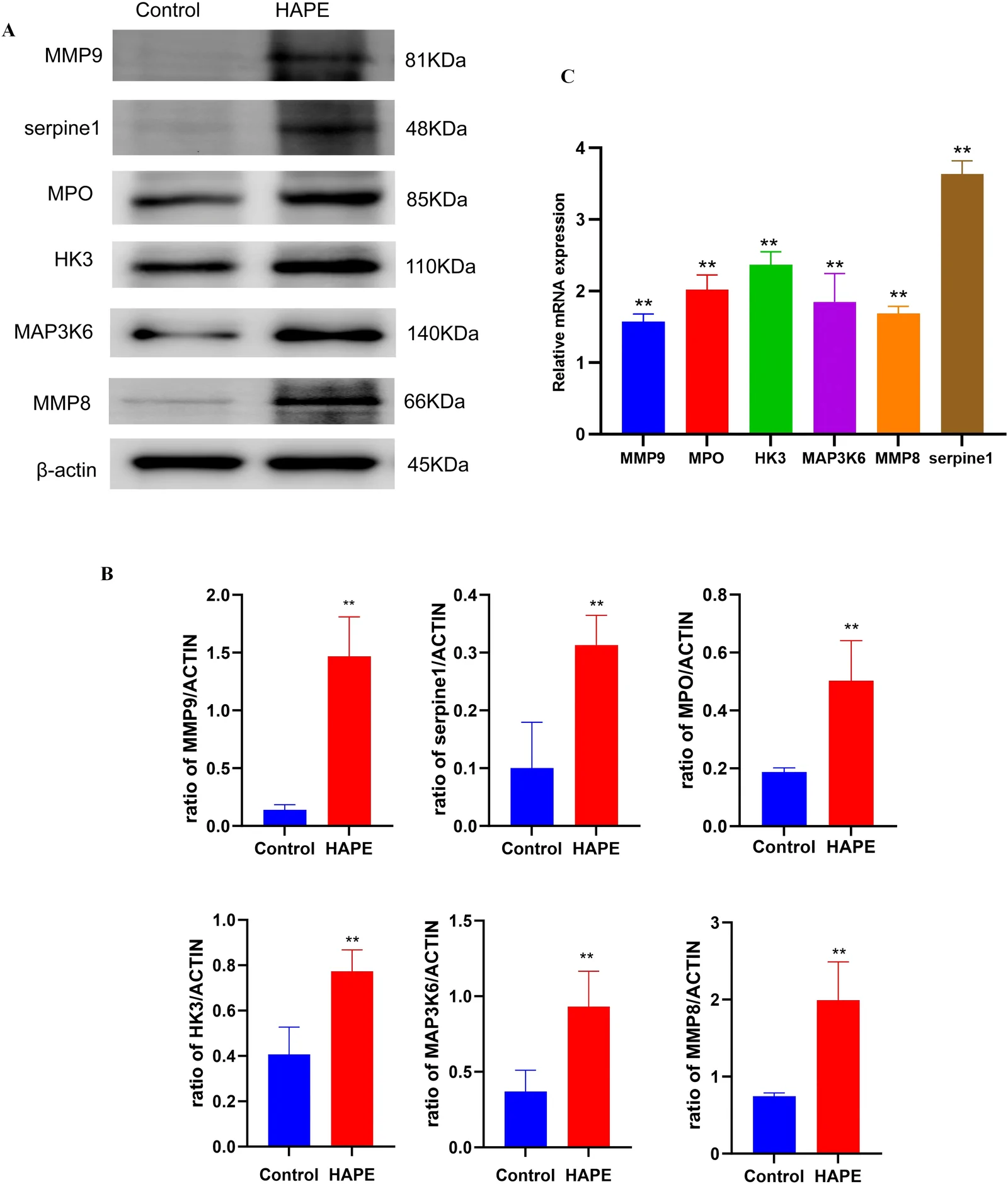
Western blot validation of candidate proteins and RT-qPCR analysis of the corresponding genes in lung tissues. **(A)** Representative Western blot bands of MMP9, MMP8, Serpine1, MPO, HK3, and MAP3K6 in the control and HAPE groups. **(B)** Densitometric quantification of Western blot bands. Protein expression was normalized to β-actin. Data are presented as mean ± SD of three biological replicates per group (n = 3 rats per group). **(C)** Relative mRNA expression levels of MMP9, MMP8, Serpine1, MPO, HK3, and MAP3K6. Gene expression was normalized to β-actin and calculated using the 2^−ΔΔCt method, with the mean value of the control group set to 1. Data are presented as mean ± SD of three biological replicates per group (n = 3 rats per group), with each biological sample measured in technical triplicate. Statistical comparisons were performed using an unpaired two-tailed Student’s t-test based on the biological replicates. ^**^P < 0.01 vs. control.

### 3.7 RT-qPCR analysis

To investigate whether the observed protein-level changes reflected transcriptional regulation, the mRNA expression of the six candidate genes was quantified by RT-qPCR (**Figure 7C**). Relative to the control group (normalized to 1), the mRNA levels of all six genes were significantly upregulated in the HAPE group (P < 0.01): MMP9 (~1.5-fold), MPO (~2.0-fold), HK3 (~2.4-fold), MAP3K6 (~1.9-fold), MMP8 (~1.7-fold), and Serpine1 (~3.6-fold). The direction of the mRNA changes was consistent with the protein-level findings, suggesting that the upregulation of these candidates is at least partly regulated at the transcriptional level. Amplification curves and melting curves confirming amplification specificity are shown in **Supplementary Figures S1**, **S2**.

### 3.8 Immunohistochemical analysis

To confirm the proteomic and Western blot findings at the tissue level and to determine the spatial distribution of the candidate proteins, immunohistochemical staining of lung sections was performed (**Figure 8**). Compared with the control group, the HAPE group exhibited markedly stronger positive staining, characterized by brown-yellow granules, for all six markers, including MMP9, MPO, Serpine1, HK3, MMP8, and MAP3K6. The staining was mainly localized in the alveolar septa, alveolar epithelial cells, and the cytoplasm of infiltrating inflammatory cells. Semi-quantitative H-score analysis confirmed that the expression levels of all six proteins were significantly higher in the HAPE group than in the control group (^**^P < 0.01; **Figure 8**), which was consistent with the proteomic and Western blot results.

**Figure 8:**
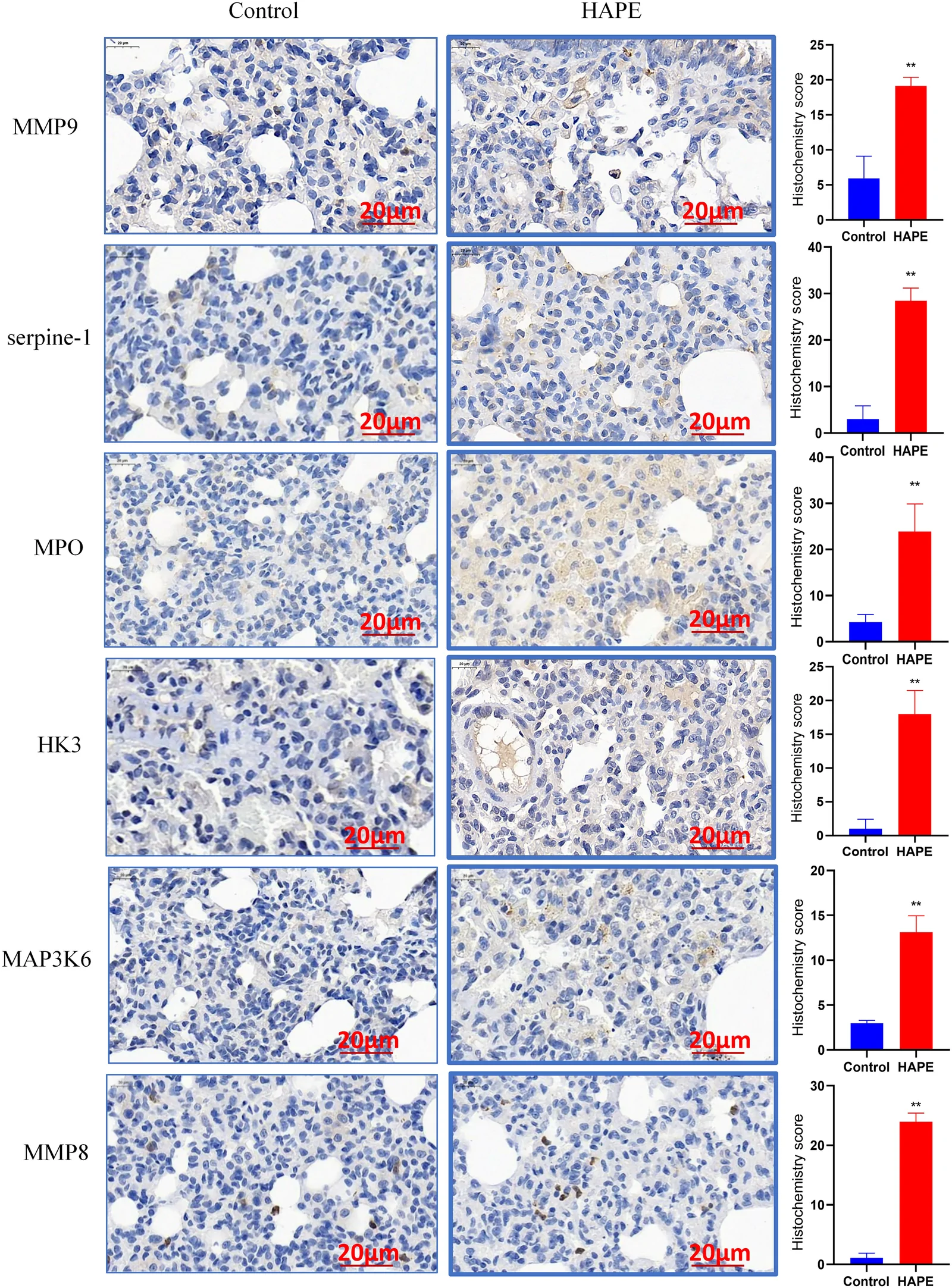
Immunohistochemical analysis of candidate proteins in lung tissues. Representative immunohistochemical staining for MMP9, MPO, Serpine1, HK3, MMP8, and MAP3K6 in the control and HAPE groups (scale bar 20 μm), together with semi-quantitative histochemistry score (H-score; staining intensity × percentage of positive cells) bar graphs. Positive staining (brown-yellow granules) was mainly localized to the alveolar septa, alveolar epithelial cells, and the cytoplasm of infiltrating inflammatory cells. Data are presented as mean ± SD (n = 3 per group), compared using unpaired two-tailed Student’s t-test (GraphPad Prism 9.0). *P < 0.05, **P < 0.01 vs. control.

## 4 Discussion

In the present study, we combined simulated high-altitude hypobaric hypoxia with treadmill exercise to establish a rat model of HAPE that recapitulates key pathological features of the human disease, including increased lung W/D ratio, elevated BALF protein, interstitial pulmonary edema, and heightened inflammatory cytokine levels. LC-MS/MS-based proteomic profiling of lung tissue identified 288 DEPs (160 upregulated and 128 downregulated), and the integrated bioinformatics analyses, together with multi-level validation, delineated four major molecular events associated with HAPE pathogenesis: neutrophil-driven inflammatory infiltration, MMP-mediated ECM degradation, Serpine1-mediated fibrinolysis inhibition, and hypoxia-associated glycolytic reprogramming.

PPI network analysis identified MMP9 as the highest-degree hub protein among the DEPs, a finding supported by its significant upregulation at the protein, tissue, and mRNA levels. MMP9 can degrade basement membrane components and tight-junction proteins such as ZO-1 and occludin, thereby disrupting the alveolar-capillary barrier and promoting pulmonary edema (Uysal and Uzun, 2019; Lo et al., 2018). Consistent with a contributory role in lung injury, doxycycline has been shown to alleviate paraquat-induced acute lung injury by inhibiting neutrophil-derived MMP9 (Zhang et al., 2019). Nevertheless, because the present study is observational in nature, the hub position of MMP9 should be interpreted as an association rather than a proven mechanistic requirement, and MMP9 is therefore best regarded as a candidate biomarker and potentially relevant mediator of HAPE rather than an established causal driver. MMP8, another neutrophil-associated collagenase that was co-upregulated in our dataset, may act in concert with MMP9 to promote ECM remodeling.

MPO is a hallmark enzyme of neutrophil activation and was significantly upregulated in HAPE lung tissue. MPO can injure the pulmonary endothelium through both enzymatic mechanisms (generation of hypochlorous acid and other reactive oxidants) and non-enzymatic mechanisms, including modulation of inflammatory cell signaling and endothelial function (Kargapolova et al., 2021; Lin et al., 2024; Siraki, 2021). The enhanced immunohistochemical staining of MPO in alveolar septa and infiltrating inflammatory cells is consistent with neutrophil accumulation in the edematous lung, supporting the notion that neutrophil-driven oxidative stress is associated with HAPE pathogenesis.

Serpine1 (PAI-1) was among the most strongly upregulated candidates in this study (approximately 3.6-fold at the mRNA level). By inhibiting tissue-type and urokinase-type plasminogen activators (tPA/uPA), PAI-1 suppresses fibrinolysis and favors fibrin deposition and microthrombosis in the pulmonary microcirculation; it can also promote cellular senescence via the p53-p21-Rb pathway and a senescence-associated secretory phenotype (SASP) that amplifies inflammation (Shaikh et al., 2024; Jiang et al., 2017; Poole et al., 2017). Pharmacological inhibition of PAI-1 (e.g., by TM5441) has been explored in related lung injury settings, suggesting that Serpine1 may represent a potentially relevant mediator worthy of functional investigation in HAPE.

HK3, a hexokinase family member, was significantly upregulated in HAPE lung tissue, indicating glycolytic reprogramming under hypoxic stress. Enhanced glycolysis is increasingly recognized as a contributor to inflammatory lung injury and lung disease progression (Yang et al., 2024; Dong et al., 2021; Yu et al., 2024; Panpan et al., 2025), and inhibition of glycolysis (e.g., with 2-deoxy-D-glucose) has shown benefit in experimental models. Our identification of HK3 in the HAPE lung proteome adds to the limited evidence linking hexokinase isoforms to high-altitude lung injury.

MAP3K6 was also upregulated in the HAPE group. MAP3K6 (apoptosis signal-regulating kinase 2, ASK2) is a member of the MAP3K family and functions upstream of the p38 and JNK MAPK cascades, coupling cellular stress sensing to downstream MAPK activation. p38 MAPK signaling is known to mediate inflammatory responses, including TIRAP-dependent activation of inflammatory cascades (Rajpoot et al., 2022), and pharmacological blockade of p38 MAPK with SB203580 has been shown to inhibit M1 macrophage polarization and attenuate LPS-induced lung injury (Li et al., 2020). Given this upstream–downstream relationship, the upregulation of MAP3K6 observed here may feed into the p38/JNK MAPK cascades implicated by these studies. Conversely, MAPK pathways can also exert protective roles against oxidative stress in certain contexts (Kumar et al., 2021), suggesting a complex, context-dependent role of MAPK signaling in HAPE that merits further study.

A strength of the present study is the concordance of results across multiple experimental layers. The upregulation of all six candidates was consistently observed in the proteomic screen, by Western blotting, by immunohistochemistry (which additionally localized the proteins to alveolar septa, alveolar epithelium, and infiltrating inflammatory cells), and at the mRNA level by RT-qPCR. This protein–tissue localization–mRNA consistency strengthens the reliability of the candidates as biomarkers and suggests that their upregulation is, at least in part, regulated at the transcriptional level.

## 5 Novelty in the context of previous studies

Although alterations of MMP9, Serpine1, and MPO in HAPE or hypoxic settings have been reported in blood-based and single-molecule studies (Singh et al., 2025; Zhang et al., 2024)—and, separately, single nucleotide polymorphisms in hypoxia-related genes have been associated with genetic susceptibility to HAPE (Wu et al., 2015), although that study did not examine the expression of these molecules—the present work provides, to our knowledge, one of the first global proteomic characterizations of lung tissue in a combined hypobaric hypoxia plus exercise rat model of HAPE. By profiling the directly affected organ, validating candidates across complementary methodological layers, identifying less-explored molecules (HK3, MAP3K6), and constructing a PPI network highlighting MMP9 as a candidate hub, this study extends prior observations and provides a resource for future mechanistic and biomarker research.

## 6 Limitations and future directions

Several limitations of this study should be acknowledged. First, only male rats were used. Sex hormones are known to modulate inflammatory and hypoxic responses, and the applicability of our findings to females remains to be determined. Second, functional validation through pharmacological inhibition or genetic modulation was not performed in this study; the identified proteins should therefore be regarded as candidate biomarkers and potentially relevant mediators rather than established causal drivers. Future studies will employ MMP9 inhibitors or knockdown approaches, as well as interventions targeting MPO, Serpine1, HK3, and MAP3K6 signaling, to test causality. Third, the key DEPs selected for validation were all upregulated, and representative downregulated proteins were not experimentally validated; future work will validate representative downregulated DEPs. Fourth, DEP screening used unadjusted P values combined with fold-change thresholds without multiple-testing correction, which is a common exploratory strategy but may increase the risk of false-positive findings; the results should thus be interpreted as hypothesis-generating.

Looking forward, emerging spatially resolved omics technologies, such as exemplified by recent advances in spatial metabolomics (Zhu, 2025), could be applied to verify the spatial distribution of the candidate molecular events identified here within the edematous lung. In addition, organ-on-a-chip platforms, such as lung-on-a-chip systems that recapitulate the alveolar-capillary interface under controlled hypoxia (Wang et al., 2024), may provide complementary human-relevant models for functional validation of the candidate biomarkers and for preclinical evaluation of potential interventions. Furthermore, natural products with anti-inflammatory and antioxidant activities may offer potential therapeutic strategies for HAPE. Studies on albiflorin and marine traditional Chinese medicine-derived compounds provide useful references for multi-target intervention (Li et al., 2025; Lu et al., 2025), although their effects on HAPE-related pathways require further validation.

## 7 Conclusion

In this study, we established a rat model of HAPE by combining simulated high-altitude hypobaric hypoxia with treadmill exercise and performed LC-MS/MS-based proteomic profiling of lung tissue. We identified 5,250 proteins and 288 DEPs (160 upregulated and 128 downregulated), which were enriched in immune/inflammatory responses, extracellular regions, glycerophospholipid metabolism, phosphatidylinositol signaling, and cell adhesion molecules. Multi-level validation by Western blotting, immunohistochemistry, and RT-qPCR consistently demonstrated upregulation of MMP9, MMP8, Serpine1 (PAI-1), MPO, HK3, and MAP3K6, and PPI network analysis highlighted MMP9 as the highest-degree hub protein. These findings delineate molecular events associated with HAPE pathogenesis and nominate candidate biomarkers and potentially relevant mediators for future functional and translational investigation.

## Data Availability

The original contributions presented in the study are publicly available. This data can be found in the iProX database with the accession number IPX0019075000.
