# Supplementary material for: Differential proteomic analysis of lung tissues in rats with high-altitude pulmonary edema and screening of potential biomarkers
Source: Front Physiol. 2026 Sep 14;17:1894550. doi: 10.3389/fphys.2026.1894550 (PMC13616702; doi:10.3389/fphys.2026.1894550)
Supplement: Supplementary file 1 [file SupplementaryFile1.docx]

**Supplementary Table S1. Representative differentially expressed proteins (DEPs) with fold changes and P values**

| **No.** | **Accession** | **Gene name** | **Protein description** | **FC (HAPE/Control)** | **Log2FC (HAPE/Control)** | **P value (HAPE/Control)** | **Regulation** | **Validated (WB/IHC/RT-qPCR)** |
| --- | --- | --- | --- | --- | --- | --- | --- | --- |
| 1 | A0A8I5ZR53 | MAP3K6 | Mitogen-activated protein kinase kinase kinase 6 | 51.380 | 5.683 | < 0.0001 (software-reported as 0) | up | Yes |
| 2 | Q31269 | RT1-Da | — | 34.260 | 5.098 | *NA* | up | No |
| 3 | A0A8I6GM59 | Hspa13 | — | 34.260 | 5.098 | *NA* | up | No |
| 4 | A0A8L2QRC0 | — | — | 34.260 | 5.098 | *NA* | up | No |
| 5 | A0A0G2JST6 | HK3 | Hexokinase OS=Rattus norvegicus | 4.845 | 2.276 | 0.00631 | up | Yes |
| 6 | A0A0H2UHI5 | serpine1 | Plasminogen activator inhibitor 1 (PAI-1) | 4.415 | 2.142 | 0.03955 | up | Yes |
| 7 | G3V7D0 | MMP8 | Matrix metallopeptidase 8 | 4.086 | 2.031 | 0.00039 | up | Yes |
| 8 | A0A0G2K1A2 | MPO | Myeloperoxidase | 3.781 | 1.919 | 0.00069 | up | Yes |
| 9 | D3ZYK8 | MMP9 | Matrix metalloproteinase-9 | 3.715 | 1.893 | 0.00038 | up | Yes |
| 10 | Q95459 | — | — | 3.042 | 1.605 | *NA* | up | No |
| 11 | A0A8I5XFF7 | Ctsg | — | 2.973 | 1.572 | *NA* | up | No |
| 12 | G3V8L7 | Itgam | — | 2.462 | 1.300 | *NA* | up | No |
| 13 | Q6P506 | — | — | 1.800 | 0.848 | *NA* | up | No |
| 14 | A0A8L2RAM6 | Hspa1 | — | 1.533 | 0.616 | *NA* | up | No |
| 15 | Q95575 | RT1-DMb | — | 0.665 | -0.588 | *NA* | down | No |
| 16 | R9PXY3 | Plcb1 | — | 0.649 | -0.623 | *NA* | down | No |
| 17 | P29826 | RT1-Bb | — | 0.605 | -0.725 | *NA* | down | No |
| 18 | P06686 | — | — | 0.583 | -0.778 | *NA* | down | No |
| 19 | D3ZMQ3 | Itga4 | — | 0.581 | -0.783 | *NA* | down | No |
| 20 | Q8VI32 | RT1-Ba | — | 0.579 | -0.787 | *NA* | down | No |
| 21 | A0A0G2K090 | Kdr | — | 0.540 | -0.888 | *NA* | down | No |
| 22 | D3ZWZ1 | Itgax | — | 0.482 | -1.052 | *NA* | down | No |
| 23 | Q6IE58 | Mcpt8l2 | — | 0.213 | -2.231 | *NA* | down | No |
| 24 | G3V839 | Pik3cb | — | 1.00E-05 | -16.610 | *NA* | down | No |
| 25 | D3ZLE6 | RT1-CE7 | — | 1.00E-05 | -16.610 | *NA* | down | No |
| 26 | F1LQT1 | Adcy7 | — | 1.00E-05 | -16.610 | *NA* | down | No |
| 27 | Q5DKU0 | RT1-Db1 | — | 1.00E-05 | -16.610 | *NA* | down | No |
| 28 | A0A0G2K1E1 | RT1-CE10 | — | 1.00E-05 | -16.610 | *NA* | down | No |
| 29 | A0A0G2K344 | Pik3ca | — | 1.00E-05 | -16.610 | *NA* | down | No |
| 30 | A0A2P1NRY9 | RT1-A2 | — | 1.00E-05 | -16.610 | *NA* | down | No |
| **No.** | **Accession** | **Gene name** | **Protein description** | **FC (HAPE/Control)** | **Log2FC (HAPE/Control)** | **P value (HAPE/Control)** | **Regulation** | **Validated (WB/IHC/RT-qPCR)** |
| 1 | A0A8I5ZR53 | MAP3K6 | Mitogen-activated protein kinase kinase kinase 6 | 51.380 | 5.683 | 0 (software-reported) | up | Yes |
| 2 | Q31269 | RT1-Da | — | 34.260 | 5.098 | *NA* | up | No |
| 3 | A0A8I6GM59 | Hspa13 | — | 34.260 | 5.098 | *NA* | up | No |
| 4 | A0A8L2QRC0 | — | — | 34.260 | 5.098 | *NA* | up | No |
| 5 | A0A0G2JST6 | HK3 | Hexokinase OS=Rattus norvegicus | 4.845 | 2.276 | 0.00631 | up | Yes |
| 6 | A0A0H2UHI5 | serpine1 | Plasminogen activator inhibitor 1 (PAI-1) | 4.415 | 2.142 | 0.03955 | up | Yes |
| 7 | G3V7D0 | MMP8 | Matrix metallopeptidase 8 | 4.086 | 2.031 | 0.00039 | up | Yes |
| 8 | A0A0G2K1A2 | MPO | Myeloperoxidase | 3.781 | 1.919 | 0.00069 | up | Yes |
| 9 | D3ZYK8 | MMP9 | Matrix metalloproteinase-9 | 3.715 | 1.893 | 0.00038 | up | Yes |
| 10 | Q95459 | — | — | 3.042 | 1.605 | *NA* | up | No |
| 11 | A0A8I5XFF7 | Ctsg | — | 2.973 | 1.572 | *NA* | up | No |
| 12 | G3V8L7 | Itgam | — | 2.462 | 1.300 | *NA* | up | No |
| 13 | Q6P506 | — | — | 1.800 | 0.848 | *NA* | up | No |
| 14 | A0A8L2RAM6 | Hspa1 | — | 1.533 | 0.616 | *NA* | up | No |
| 15 | Q95575 | RT1-DMb | — | 0.665 | -0.588 | *NA* | down | No |
| 16 | R9PXY3 | Plcb1 | — | 0.649 | -0.623 | *NA* | down | No |
| 17 | P29826 | RT1-Bb | — | 0.605 | -0.725 | *NA* | down | No |
| 18 | P06686 | — | — | 0.583 | -0.778 | *NA* | down | No |
| 19 | D3ZMQ3 | Itga4 | — | 0.581 | -0.783 | *NA* | down | No |
| 20 | Q8VI32 | RT1-Ba | — | 0.579 | -0.787 | *NA* | down | No |
| 21 | A0A0G2K090 | Kdr | — | 0.540 | -0.888 | *NA* | down | No |
| 22 | D3ZWZ1 | Itgax | — | 0.482 | -1.052 | *NA* | down | No |
| 23 | Q6IE58 | Mcpt8l2 | — | 0.213 | -2.231 | *NA* | down | No |
| 24 | G3V839 | Pik3cb | — | 1.00E-05 | -16.610 | *NA* | down | No |
| 25 | D3ZLE6 | RT1-CE7 | — | 1.00E-05 | -16.610 | *NA* | down | No |
| 26 | F1LQT1 | Adcy7 | — | 1.00E-05 | -16.610 | *NA* | down | No |
| 27 | Q5DKU0 | RT1-Db1 | — | 1.00E-05 | -16.610 | *NA* | down | No |
| 28 | A0A0G2K1E1 | RT1-CE10 | — | 1.00E-05 | -16.610 | *NA* | down | No |
| 29 | A0A0G2K344 | Pik3ca | — | 1.00E-05 | -16.610 | *NA* | down | No |
| 30 | A0A2P1NRY9 | RT1-A2 | — | 1.00E-05 | -16.610 | *NA* | down | No |

Notes: DEPs were defined as unadjusted P ≤ 0.05 with FC ≥ 1.5 (upregulated) or FC ≤ 0.67 (downregulated); unpaired two-tailed Student's t-test; n = 3 per group.

Adjusted P values are not available: exploratory DEP screening used unadjusted P values without multiple-testing correction (see manuscript Sections 2.4 and 5).

Rows highlighted in blue are the six candidate proteins validated by Western blotting, immunohistochemistry and RT-qPCR.

NA = P value not available in the current source export (chord-diagram data provide Log2FC only); FC for these entries was computed as 2^Log2FC.

30 of 288 DEPs are currently listed.

**Supplementary Figure S1.** Amplification curves of the RT-qPCR assays for the six candidate genes and β-actin.

**Supplementary Figure S2.** Melting curves of the RT-qPCR assays, each showing a single peak, confirming amplification specificity.
